# Trust Performance Indicators and Trust Stress Tests: A Conceptual Proposition for Trustworthy Health Data Spaces

**DOI:** 10.3389/ijph.2025.1608708

**Published:** 2025-10-16

**Authors:** Felix Gille, Paola Daniore, Laura Maaß, Federica Zavattaro

**Affiliations:** ^1^ Digital Society Initiative (DSI), University of Zurich, Zurich, Switzerland; ^2^ Institute for Implementation Science in Health Care (IfIS), University of Zurich, Zurich, Switzerland; ^3^ Center for Digital Trust, Swiss Federal Institute of Technology Lausanne (EPFL), Lausanne, Switzerland; ^4^ Leibniz ScienceCampus Digital Public Health Bremen, Bremen, Germany; ^5^ SOCIUM Research Center on Inequality and Social Policy, University of Bremen, Bremen, Germany

**Keywords:** trust, evaluation, health data space, performance indicator, stress testing

## Abstract

The development of trustworthy Health Data Spaces (HDS) is currently in the spotlight of digital health policy. Diverse stakeholders agree on the importance of trust for the adoption and legitimacy of HDS. This emphasis on trust has led to the development of conceptual work describing what trust in HDS entails, along with initial suggestions on how trust principles can be operationalized in HDS governance and architecture. In contrast, little research has been conducted on methods to evaluate the performance of trust-building principles and the overall trustworthiness of HDS. In response, we propose two distinct methodologies that share a common focus on assessing trustworthiness: A) Trust Performance Indicators collect routine data related to trust-building principles. B) Trust Stress Tests support the design of resilient HDS architectures by identifying potential future scenarios that could undermine their trustworthiness. Through these methodologies, we aim to contribute to the ongoing development of trustworthy HDS.

## Introduction

Trust is climbing the policy agenda. Among others, the European Observatory on Health Systems and Policies has placed trust in the spotlight of health system governance, policy-making, and stakeholder discussions. Trust is the foundation of and a key enabler for the digital transformation of health systems [1]. The importance of trust is rooted in its ability to increase acceptance and support of complex health system activities [2]. A prominent example in the context of Switzerland, and a parallel development to the European Health Data Space (EHDS), is the introduction of a Swiss health data space (SHDS) [3]. Politicians, researchers, and other stakeholders alike have identified trust as a key enabler for the legitimate introduction of data spaces [4].

Outside of health systems, there is growing interest in the role of trust across various contexts. For example, the concept of ‘digital trust’ has been coined in digital consumer services, such as online banking and e-commerce [5–7]. Similarly, trust in science has become a subject of debate in the context of climate change, which is becoming increasingly influenced by the observed political shift towards the right wing. This has translated into prevalent societal unease, divisions, and questions of the government’s trustworthiness in some countries [5, 6, 8]. Issues of trust are evidently topical across different nations and systems beyond the health sector, calling for broader system-thinking approaches and comprehensive methodologies to foster trust [9].

### Current Challenges in Trust Building

Without a common definition existing, trust can be understood as “ […] a bet about the future contingent actions of others” [10]. It is a relational concept where A trusts B in anticipation of a beneficial outcome. To build trust, A needs information about B’s trustworthiness. While trust is a relational concept between two or more parties, trustworthiness is a trait of the trusted party [11]. This information relates to A’s individual or collective past experiences, A’s present perceptions of B’s ability to achieve a beneficial outcome, and A’s future anticipation of what the beneficial outcome should be [2].

To make trust a workable concept for governance, policy, and health system planning, researchers developed a range of trust frameworks describing public, patient, and professionals’ trust in the health system and digital health interventions. Examples include frameworks describing public trust in health data sharing within health systems, public trust in national electronic patient record systems, or user trust in artificial intelligence applications in medicine [12–18]. Besides the existing challenges in developing comprehensive communication strategies, policies, and service system architectures that aim to build trust, difficulties in evaluating trust-building efforts remain a key inhibitor to effective trust-building. Targeted trust-building activities depend on the ability to evaluate their successes. Evaluation, including measurement, is critical to understanding the health system’s ability to build and maintain trust.

Evaluations are currently informed by survey instruments, including qualitative components and measurement scales [19]. While survey methods are informative to understand trust at the point of data collection, some of the authors argued elsewhere that such instruments are potentially a weak tool for collecting routine data about the trustworthiness of a data driven healthcare service, especially in a dynamic environment where the conceptualization of trust might not remain stable over time [20]. Due to the conceptual complexity of trust, we argue that additional methods are needed to collect data about trustworthiness and trust comprehensively. Our view resonates with other researchers calling for methodological creativity to find alternative approaches to measure trust [21].

### Applying Trust Stress Tests and Trust Performance Indicators

The limits of current trust evaluations carry the risk of generating weak evidence. Governing a health system activity based on weak evidence can potentially undermine trust-building efforts, strategic decision-making for the health system and policy design, healthcare communication, health system transformation, and the health system performance at large. Consequently, we need to elevate trust beyond a mere policy priority and actively build trust in practice, informed by up-to-date evidence and comprehensive conceptual work.

In search of novel methods to evaluate the trustworthiness of trust-building activities, we suggest the development and use of Trust Performance Indicators (TPI) and Trust Stress Tests (TST). The reasoning is that both methods are based on rigorously developed and up-to-date conceptual frameworks describing what users understand as a trustworthy health data space. Relational aspects, such as user interaction with health data spaces or user views and perceptions, can be either evaluated as part of TPIs or by employing user surveys. By employing different methodologies, TPIs and TSTs complement each other in the shared goal of evaluating the trustworthiness of a health service more comprehensively. While TPIs focus on continuous monitoring through the collection of routine data, TSTs focus on the identification of system weaknesses during selected periods, such as the design process. We anticipate that TPIs and TSTs will help to build and maintain trust in the health system, provide continuous insights on the effectiveness of trust-building activities embedded in the health service design, and allow for economic evaluations of these activities in resource-limited environments.

The concepts proposed in this article are based on our research on public trust in health data use within health systems and digital public health [2, 15, 22] and on previously published work on TPIs and TSTs [20, 23]. In this article, we focus on defining and providing examples of TPIs and TSTs for public trust in a health data space as a possible use case. The discussed ideas are applied to the SHDS as an example, but are transferable to ongoing discussions about the introduction of data spaces beyond health data internationally.

## What Is Public Trust in Health Data Sharing?

The foundation for the development of TPIs and TSTs for the SHDS, as described below, lies in a comprehensive conceptual understanding of public trust in health data sharing. TPIs and TSTs are meaningful if they are directly linked to actions that the public perceives as fostering trust in health data spaces.

The public trusts health data-sharing activities in anticipation of a net-benefit for the individual citizen, the collective society, and the health system itself [2]. Trustworthy health system designs are anticipated to increase public willingness to participate in such activities. Further, public trust legitimises health system activities, as trusted health system actors enjoy the public’s support to act in their best interest. Previous research on health data sharing activities for secondary use in NHS England and other European countries, including Switzerland, shows that members of the public associate a range of activities as essential for trust-building in the context of health data sharing [2]. Examples are clear communication strategies to increase transparency, upholding high levels of data security and privacy protection, compliance with ethics and laws, or maintaining high reliability of the data system.

Complementing the conceptual understanding, our recent qualitative study with members of the Swiss public revealed four focal application points which are particularly relevant for trust-building activities in health data spaces [24]:

a) consent management,
b) health data linkage,
c) data coordination centres, and
d) international health data exchange.

Combining knowledge about public understanding of essential characteristics of trustworthy health data flows [2] with knowledge about pivotal activities crucial for building a trustworthy health data space in practice [24] is exceptionally valuable to guide the design and application of actionable TPIs and TSTs, as outlined below.

## What Are Trust Performance Indicators?

TPIs are a set of indicators anchored in comprehensive conceptual work that describe trust-building activities in a given context [25]. Similar to other indicators such as quality, health, or key performance indicators, TPIs, in our example, are designed to a) guide health system interventions towards fostering trustworthy targets and b) collect routine data about their performance in achieving trust-building targets. These targets include activities such as maintaining high levels of data security, protecting privacy, tracing data access, communicating with the public about data use, or regulatory compliance.

Indicator sets are commonly used to assess the performance of health systems [20]. An example of a comprehensive indicator set in the context of the digital economy is the Digital Intelligence Index, comprising a Digital Trust Scorecard with 160 different indicators covering four domains: Supply Conditions, Demand Conditions, Institutional Environment, and Innovation and Change [26]. Another indicator set is the Digital Public Health Maturity Index, comprising 96 indicators across five domains: Degree of Application, Legal Framework, Social Willingness and Capacity, DiPH Tools, and Information-Communication-Technology requirements [22]. Yet, a wide-ranging indicator set that focuses on trust in health data spaces is missing.

While no commonly accepted development methodology exists, we suggest applying a structured consensus-building process of the Delphi technique that involves leading experts and other stakeholder groups such as members of the public, patient group representatives, and medical professionals to develop the indicators [22]. It is critical that the development and consensus process:

a) is rooted in the conceptual framework of public trust in health data sharing,
b) targets the key activities for trust-building in the health data space, and
c) involves experts who are familiar with the data space architecture and trust research.

Since the general concept of indicators is familiar to most health system stakeholders and integral to performance analysis within many healthcare institutions and companies, TPIs may be developed based on existing indicators that are fit for purpose. Moreover, the introduction of TPIs should aim to be non-disruptive and seamlessly integrated into stakeholders’ existing workflows. It will be crucial to balance a manageable number of TPIs against a comprehensive coverage of the underlying conceptual framework.

## What Are Trust Stress Tests?

Informed by a clear conceptual understanding of trust in a given context, TSTs are a set of possible health system scenarios that reflect impactful and realistic future events or activities that could undermine the trustworthiness of the health system activity in focus. The value of TSTs lies not in the scenarios themselves, but in answering the question: *How do we avoid such scenarios?* Notably, the TSTs need to target the trust-building themes that are described in the conceptual framework of trust (e.g., protection of privacy, data security, or communication) and ensure that the knowledge gained from applying TSTs is translated into the governance structure of the health system activity in focus. Unlike performance indicators, stress tests or future scenarios may not be widely recognized across different stakeholder groups. TSTs, however, will be used periodically, for example, during the design phase and structural evaluation processes of the Swiss health data space. Consequently, TSTs need to have a direct influence on the health data space architecture.

While there is no commonly accepted methodology to develop stress tests, we propose that TSTs utilise scenario planning, which explores possible future scenarios and provides insights - using quantitative, mixed-methods, and qualitative approaches–into how we can reach a potential future from today’s views or prepare for a possible future [27]. The application of TST scenarios serves two different purposes:

1. Identification: to identify weak spots in a health system activity that could erode trust
2. Resilience: to inform design improvements, ensuring the development of a health system intervention that is able to confront and overcome trust-undermining activities.

Stress testing is a well-established practice in engineering and development, applied to evaluate the stability of systems or entities, including the assessment of system breaking points beyond standard operational capacity [28]. Stress tests are used across various domains, including financial services as part of risk management practices, critical infrastructure networks such as power plants or railways, and computer systems in informatics [29, 30]. By identifying weaknesses and points of failure, stress testing generates knowledge that informs design refinements to build more resilient systems.

## Use Case: TPIs and TSTs in the Context of Health Data Spaces

The combination of TPIs and TSTs provides knowledge about the trustworthiness of a health data space. Table 1 sketches how trust-building themes can be translated into TPIs and TSTs. To link this article to present developments in data space designs, we hand-picked one trust-building activity (Identity & Attestation Management) from the Data Spaces Building Blocks published by the Data Spaces Support Center in line with our own conceptual work on trust in data sharing [15, 31]. “Identity and attestation management is foundational to onboarding participants, verifying their compliance with the Data Space Rulebook, and issuing proofs of membership that facilitate trusted data exchanges.” [32]. Positive user experience and preparedness both contribute to perceptions of trustworthiness. If both are considered in the design process of an Identity & Attestation Management, the system may be perceived as more trustworthy by its users. The sketched TPIs and TSTs showcased in Table 1 aim to facilitate initial discussions and are not to be used in practice as they are not rigourusly developed.

**TABLE 1:** Example trust performance indicators and trust stress tests (Zurich, Lausanne, Bremen, 2025).

Identity & Attestation Management				
No.	Content	Method	Target value	Explanation
TPI 1	Time of identity onboarding	Track time of onboarding	Time	Short time can be an indication of smooth operation and good user experience
TPI 2	Incident response time to identify breaches	Measure time needed to detect and response to identity breaches	Time	Fast response might reduce damage and shows preparedness
TPI 3	False positive and negative identity authorisation	Count a) incorrect granted access, and b) denied access to rightful users	Incident rate	High false rates can lead to data breaches and poor user experience. In turn low false rates indicate a well-designed process contributing to good user experience
TST	Malicious insider attack	A legitimate user uses the access rights for evil actions	Detection rate	Design systems to detect anomalies in user behaviour and signs of abuse

The sketched trust performance indicators (TPI 1, 2, 3) in Table 1 collect useful data to assess the performance of trust building activities within the activity “Identity Management”. The TPIs center around user experience and preparedness. Our trust research shows that positive user experience contributes to perceptions of trustworthiness and trust building more broadly. The link between past positive experiences and trust building is widely acknowledged in trust research and supported by our daily life experiences as users of digital technology and complex systems. Similarly, when users understand that those running digital technology have contingency plans ready do confront malicious actions, users may perceive such systems as more trustworthy.

TSTs identify future scenarios that may undermine the trustworthiness and therby help to design a more resilient Identity Management Systems for the SHDS. The TST in Table 1 has the ability to undermine the trustworthiness of the Identity Management Process and help to prepare continsency plans. A malicious insider misusing his/her legitimate access rights can undermine the trustworthiness of the overall system with evil actions potentially harming a wide range of users.

## Future Research and Development

We propose TPIs and TSTs as valuable methods for the evaluation of the trustworthiness of health data spaces in Switzerland, the European Union and beyond. We also find value in their use to guide the design of a robust governance framework that is capable of anticipating and setting safeguards in response to future trust-undermining activities. Together, they offer a new and complementary approach to conventional survey methods to collect evidence about the trustworthiness of health system activities.

We anticipate that, following development and rigorous field testing, the successful implementation and knowldge about trustworthiness gained from TPIs and TSTs will hinge on stakeholder support. The participation of a wide network of actors, including public administration, industry, researchers, and others, contributes to the success of a health data space. In turn, the uptake of TPIs and TSTs will depend on the active engagement of these stakeholders by committing resources. Aligned interests, together with understanding the clear benefit of high levels of trust and how TPIs and TSTs can contribute to achieving such levels, will be vital. Linking trust performance to health system actors’ accountability represents a way to drive action, as suggested in the context of building digital trust in the Swiss industry. Executives in charge of the health data space activities may act as role models, setting the tone for others to follow [33]. Lastly, as trust is a dynamic concept that evolves with societal changes over time, both TPIs and TSTs need to be periodically updated to reflect up-to-date public perceptions of trustworthiness.

We invite others to discuss and test in practice the ideas presented in this paper and to build and maintain trustworthy data spaces in healthcare and beyond. It will be important to conduct field testing beyond the European region as data spaces and data exchange are intended to function globally. TPIs and TSTs offer, in our opinion, a viable way forward to support the trustworthy transformation of health systems and the design of trustworthy data space architectures.
